# Supplementary material for: AHL-differential quorum sensing regulation of amino acid metabolism in Hafnia alvei H4
Source: Microbiol Spectr. 2024 Feb 23;12(4):e00687-23. doi: 10.1128/spectrum.00687-23 (PMC10986605; doi:10.1128/spectrum.00687-23)
Supplement: Supplemental material — Figures S1 to S4. [file spectrum.00687-23-s0001.docx]

**Supplementary Information**


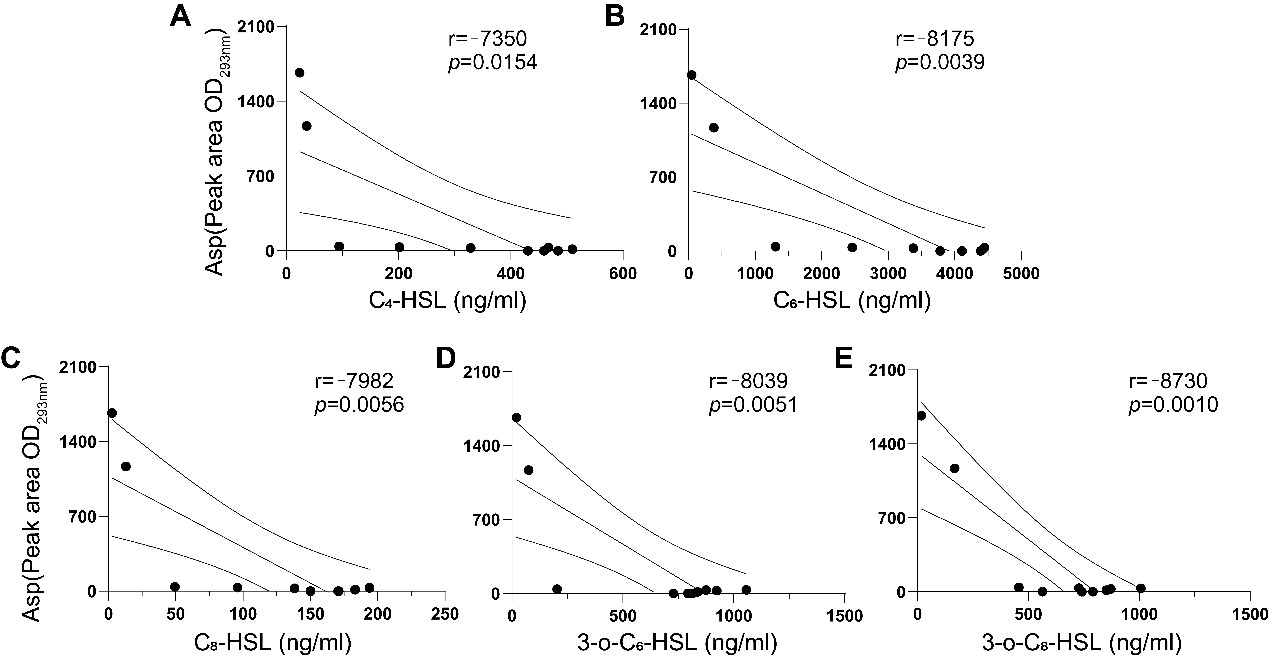


**Fig. S1** The correlations of aspartate with C4-HSL (A), C6-HSL (B), C8-HSL (C), 3OC6-HSL (D), and 3OC8-HSL (E), respectively.


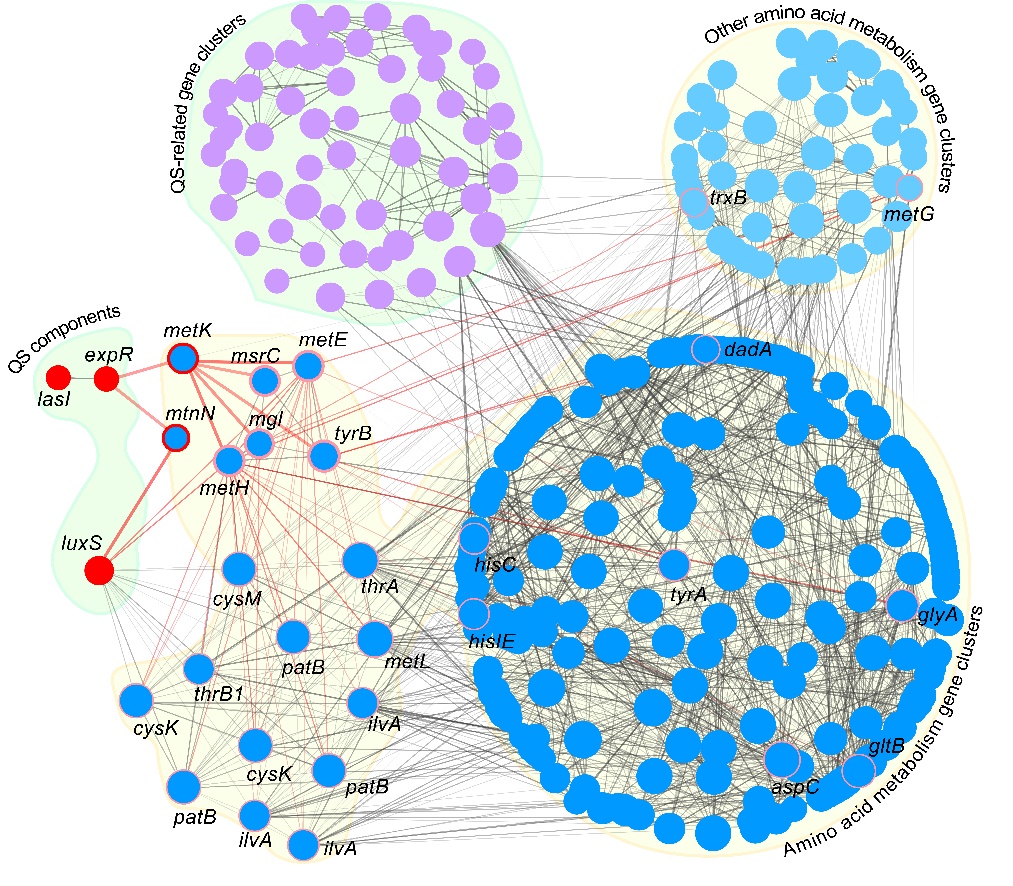


**Fig. S2** The protein interaction network between QS gene cluster and amino acid metabolic gene cluster obtained from genome of *H. alvei* H4.


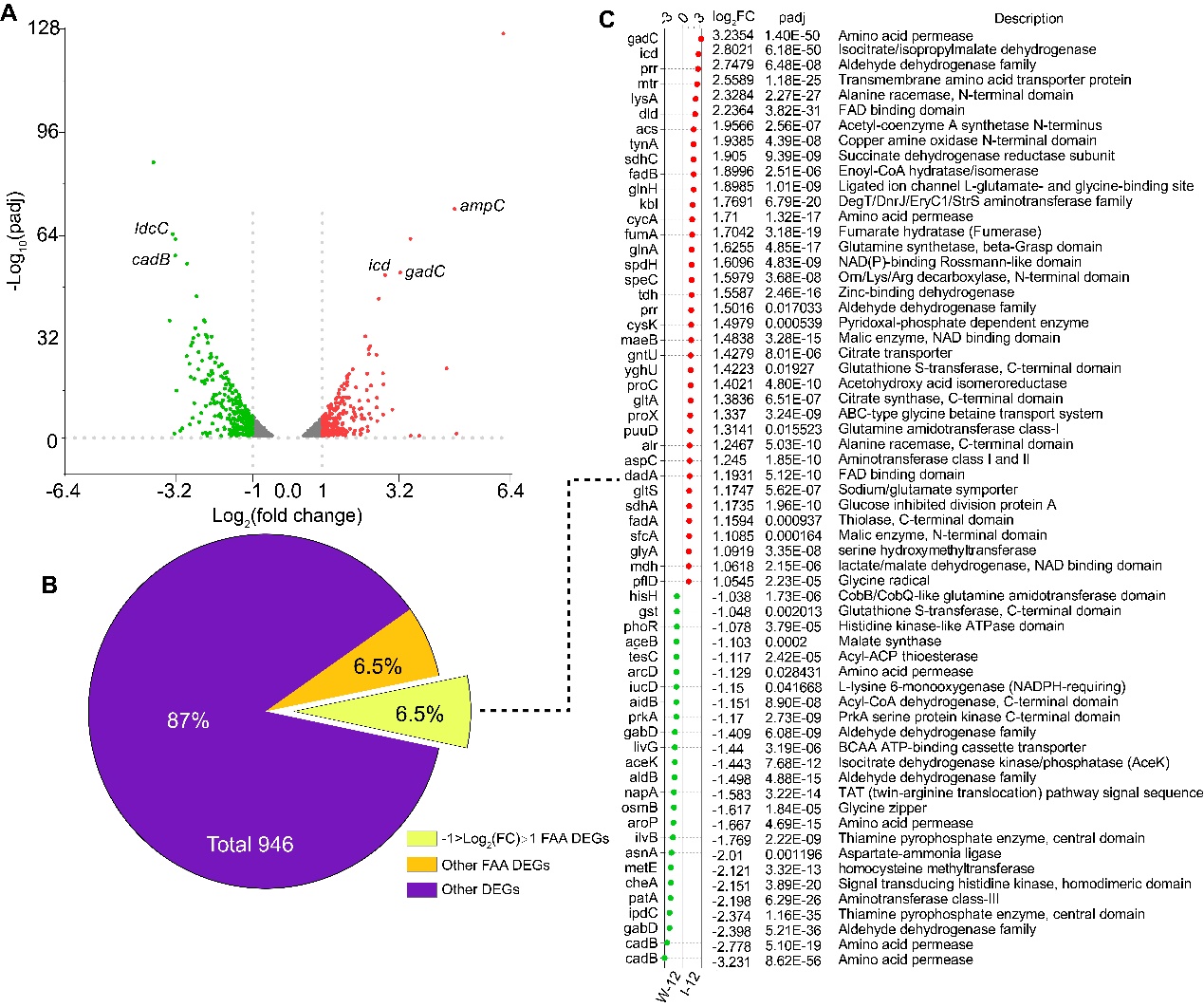


**Fig. S3** Comparative transcriptome analysis between WT and Δ*luxI*, including volcano plot of gene expression data (A), Pie charts illustrating the relative percentages of amino acid genes regulated by QS (B), and amino acid metabolic genes with log2 FC below -1 and above 1 (C).


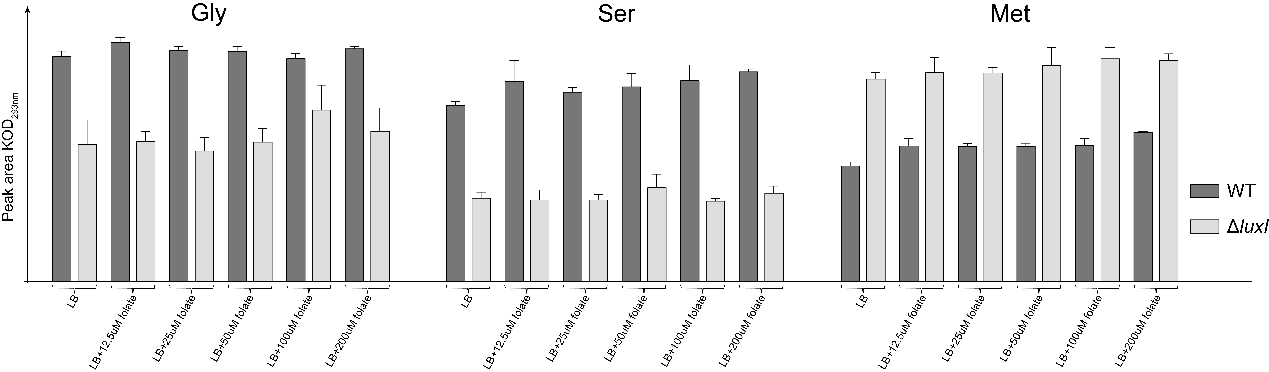


**Fig. S4** The effects of different concentrations of folate on the accumulation of glycine, serine, and methionine in WT and Δ*luxI*, respectively.
